# The Tumor Risk Score (TRS) – next level risk prediction in head and neck tumor surgery

**DOI:** 10.1007/s10006-024-01281-8

**Published:** 2024-07-20

**Authors:** Anne Klausing, Kristina Waschk, Frederick Far, Markus Martini, Franz-Josef Kramer

**Affiliations:** 1 Department of Maxillofacial and Plastic Surgery, University Hospital Bonn, Bonn, Germany; 2 Department of Internal Medicine, Spital Männedorf, Männedorf, Switzerland; 3 Department of Maxillofacial and Plastic Surgery, Kliniken Mettmann-Süd St. Josefs Krankenhaus, Hilden, Germany

**Keywords:** Head and neck cancer, Risk prediction, Resource allocation, Intensive care, Risk index, Charlson comorbidity index

## Abstract

**Purpose:**

Head and neck cancer surgery often requires postoperative monitoring in an intensive care unit (ICU) or intermediate care unit (IMC). With a variety of different risk scores, it is incumbent upon the investigator to plan a risk-adapted allocation of resources. Tumor surgery in the head and neck region itself offers a wide range of procedures in terms of resection extent and reconstruction methods, which can be stratified only vaguely by a cross-disciplinary score. Facing a variety of different risk scores we aimed to develop a new Tumor Risk Score (TRS) enabling anterograde preoperative risk evaluation, resource allocation and optimization of cost and outcome measurements in tumor surgery of the head and neck.

**Methods:**

A collective of 547 patients (2010–2021) with intraoral tumors was studied to develop the TRS by grading the preoperative tumor size and location as well as the invasiveness of the planned surgery by means of statistical modeling. Two postoperative complications were defined: (1) prolonged postoperative stay in IMC/ICU and (2) prolonged total length of stay (LOS). Each parameter was analyzed using TRS and all preoperative patient parameters (age, sex, preoperative hemoglobin, body-mass-index, preexisting medical conditions) using predictive modeling design. Established risk scores (Charlson Comorbidity Index (CCI), American Society of Anesthesiologists risk classification *(*ASA), Functional Comorbidity Index (FCI)) and Patient Clinical Complexity Level (PCCL) were used as benchmarks for model performance of the TRS.

**Results:**

The TRS is significantly correlated with surgery duration (*p* < 0.001) and LOS (*p* = 0.001). With every increase in TRS, LOS rises by 9.3% (95%CI 4.7–13.9; *p* < 0.001) or 1.9 days (95%CI 1.0-2.8; *p* < 0.001), respectively. For each increase in TRS, the LOS in IMC/ICU wards increases by 0.33 days (95%CI 0.12–0.54; *p* = 0.002), and the probability of an overall prolonged IMC/ICU stay increased by 32.3% per TRS class (*p* < 0.001). Exceeding the planned IMC/ICU LOS, overall LOS increased by 7.7 days (95%CI 5.35–10.08; *p* < 0.001) and increases the likelihood of also exceeding the upper limit LOS by 70.1% (95%CI 1.02–2.85; *p* = 0.041). In terms of predictive power of a prolonged IMC/ICU stay, the TRS performs better than previously established risk scores such as ASA or CCI (*p* = 0.031).

**Conclusion:**

The lack of a standardized needs assessment can lead to both under- and overutilization of the IMC/ICU and therefore increased costs and losses in total revenue. Our index helps to stratify the risk of a prolonged IMC/ICU stay preoperatively and to adjust resource allocation in major head and neck tumor surgery.

## Introduction

Preoperative risk stratification should contribute to the avoidance or control of peri- and postoperative complications, enable demand-based allocation of resources, and ensure safe treatment. With a variety of different risk scores, it is incumbent upon the investigator to stratify a patient’s preoperative health status and plan a risk-adapted resource allocation [1–3]. One of the most common instruments is applied primarily in the anesthesiologic premedication visit – the American Society of Anesthesiologists risk classification (ASA) [4]. However, the purely patient-oriented indices remain critical due to their lack of discriminatory power with regard to the range of procedures performed in different departments [5]. Tumor surgery in the head and neck region offers a wide range of procedures in terms of resection and reconstruction procedures, which can only be stratified vaguely using a cross-disciplinary score [1]. Some discriminatory criteria, such as operative time, are only available ex-post and therefore not suitable for predictive risk evaluation and resource allocation. Precise risk adjustment is equally required for evaluation of care complexity and quality [6]. Our novel and specific Tumor Risk Score (TRS) provides a selective preoperative risk stratification tool in head and neck tumor surgery and thus enables a predictable resource allocation and optimization of cost and outcome measurements.

## Materials and methods

Data from a retrospective cohort of 547 patients with intraoral tumors from 2010 to 2021 was collected. The new Tumor Risk Score (TRS) was generated by graduating the preoperative clinical tumor size and localization as well as the invasiveness of the planned surgery using statistical regression modeling (**Table 1**). Therefore, the oral cavity was categorized into different regions (tongue, floor of the mouth, mandibular alveolar ridge, maxillary alveolar ridge, planum buccale, soft palate, arcus palatoglossus, hard palate, submandibular gland) using clinically assessed anatomical landmarks, which are routinely used in the preoperative assessment to determine the localization of the tumor. To grade the size and extent of the tumor, a categorization was based on whether it comprised 1, 2 or 3 of these regions. Additionally, we graded the invasiveness of the planned surgery categorizing the extend of the bony resection on the one hand and the extent of the reconstruction (local, regional or microvascular flap) on the other hand (Table 1).

**Table. 1:**
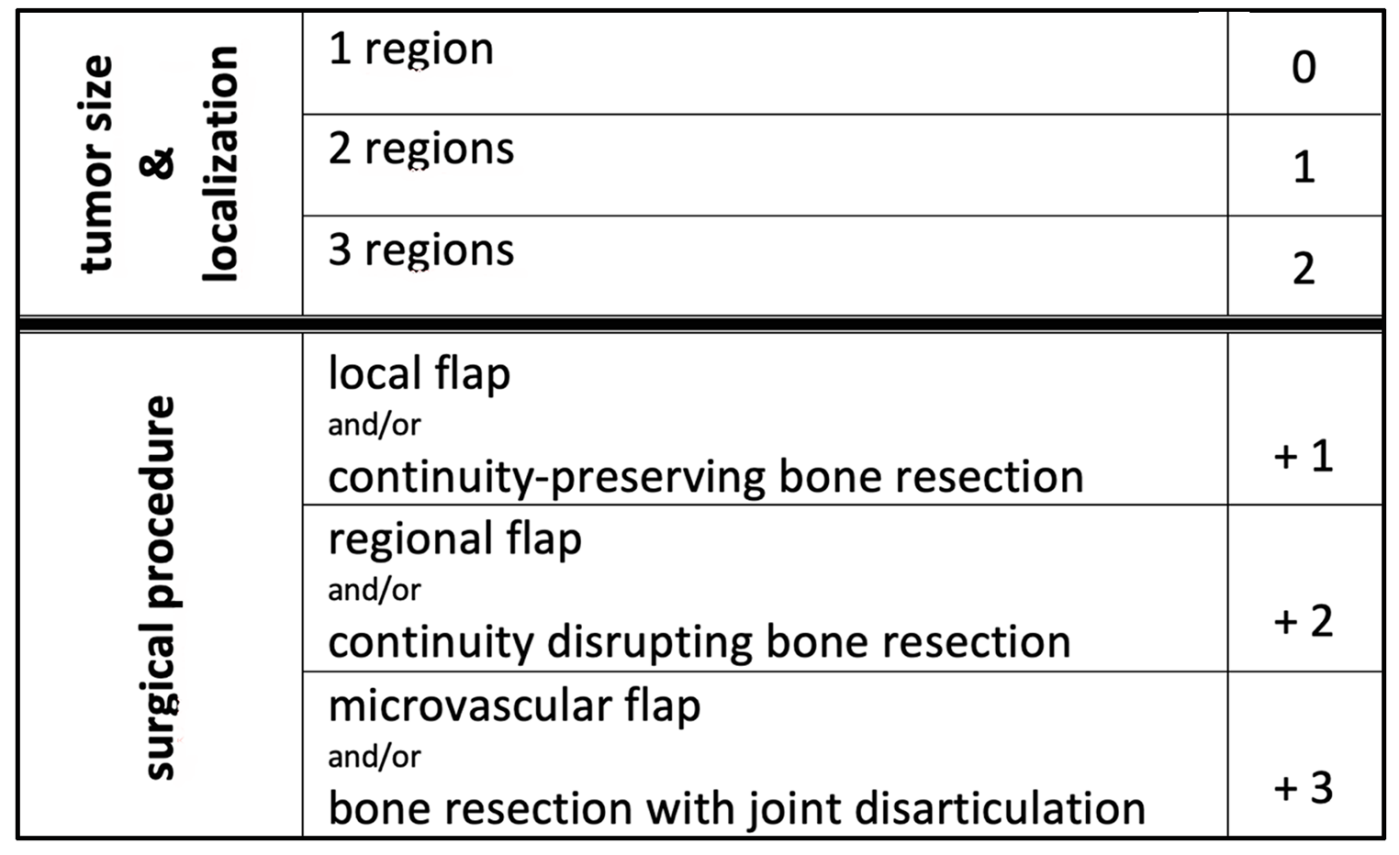
Calculation of TRS (Tumor Risk Score) using weighted scores for tumor size, localization and surgical procedure

Additionally, the preoperative patient parameters age, sex, preoperative hemoglobin (Hb) level and body mass index (BMI) were collected as potential predictors for complications based on clinical experience and literature review [7–9]. Two postoperative complications were defined: (1) prolonged postoperative stay in the intermediate care unit (IMC) or intensive care unit (ICU) and (2) prolonged total length of stay (LOS). Prolonged IMC/ICU stay was defined as > 1 day. To evaluate the length of stay according to the complexity and morbidity of the patient, the variable “long-stay patient” was defined. For this purpose, the individual case-specific upper limit LOS was assessed. This case-specific upper limit LOS is determined by the G-DRG system (German Diagnosis Related Group system, referred to as “DRG”), which is defined through a complex algorithm considering primary and secondary diagnoses on admission, the medical procedures performed and their complexity. If a patient exceeded the individual upper limit LOS according to the DRG, he was classified as “long-stay patient”. For example, a complex tumor resection procedure with microvascular reconstruction often accounts for DRG “D02A” with a lower limit LOS of 6 days and an upper limit LOS of 36 days. If a patient with DRG “D02A” exceeded a LOS of 36 days, he was characterized as a “long-stay patient”. In case a patient exceeds his individual upper limit LOS the daily revenue is no longer cost-covering. Each of the two outcome parameters was analyzed using the TRS and all preoperative patient parameters with a predictive modeling design that accounts for even moderately correlated variables. Previously established risk scores (Charlson Comorbidity Index (CCI), ASA class (ASA), Functional Comorbidity Index (FCI)) and the Patient Clinical Complexity Level (PCCL), as the overall patient-related severity score, were used as comparative benchmarks for the performance of the TRS. Exclusion criteria was defined as insufficient or missing patient data, thus three patients had to be excluded due to missing data in some or all model parameters.

Semielasticities (ey/dx, dy/dx) were used to quantify effects on the outcome variable, multiple linear and Poisson regression analyses were used as generalized linear models, coefficients were presented as odds ratios (OR) and incidence rate ratios (IRR). Correlations were reported using Spearman’s coefficient. To calculate probabilities for the postoperative outcome measures, coefficients (a) and absolute terms (b) were derived from the logit function and transferred to the exponential function. Model goodness-of-fit analysis was performed using receiver operating characteristic (ROC) curves. A p-value of 0.05 was considered statistically significant.

Statistical analysis was performed using STATA/SE 16.1 (Stata-Corp, College Station, TX, USA). The study was reviewed and approved by the local ethics committee (No. 268/17) and complies with the principles of the Declaration of Helsinki for research involving human subjects.

## Results

Data were collected on a total of 547 patients with intraoral tumors in the period 2010–2021. The gender distribution was balanced (male: *n* = 284 (51.9%), female: *n* = 263 (48.1%)). The mean age at the time of surgery was 65.4 (± 13.3) years. The tumor entity was mainly squamous cell carcinoma (*n* = 493 (90.1%)), followed by mucoepidermoid carcinoma (*n* = 14 (2.6%)) and adenocarcinoma (*n* = 11 (2.0%)). Other tumor entities included osteosarcoma (*n* = 5 (0.9%)), ameloblastoma (*n* = 3 (0.6%)), basal cell carcinoma with predominantly intraoral localization (*n* = 3 (0.6%)), giant cell granulomas (*n* = 3 (0.6%)) and other tumor entities (mucosal melanoma, pleomorphic adenoma, keratocystic odontogenic tumor, epithelial-myoepithelial carcinoma) (*n* = 14 (2.6%)). Preoperative localization were clinically defined as 1 region (tongue (*n* = 122; 22.3%), floor of the mouth (*n* = 106; 19.4%), mandibular alveolar ridge (*n* = 87; 15.9%), maxillary alveolar ridge (*n* = 60; 11.0%), planum buccale (*n* = 26; 4.8%), soft palate (*n* = 23; 4.2%), arcus palatoglossus (*n* = 14; 2.6%), hard palate (*n* = 4; 0.7%), and submandibular gland (*n* = 3; 0.6%)); 2 regions (floor of the mouth + tongue (*n* = 32; 5.9%), floor of the mouth + mandibular alveolar ridge mandible (*n* = 17; 3.1%), hard palate + soft palate (*n* = 14; 2.6%) and mandibular alveolar ridge + planum buccale (*n* = 9; 1.7%)), and 3 regions (tongue + floor of the mouth + mandibular alveolar ridge (*n* = 8; 1.5%), floor of the mouth + mandibular alveolar ridge + planum buccale (*n* = 8; 1.5%), and mandibular alveolar ridge + planum buccale + soft palate (*n* = 8; 1.5%)). The extent of tumor resection was classified into four degrees: resection without bone resection in 225 cases (41.2%), continuity-preserving bone resection in 171 cases (31.3%), bone resection with continuity interruption in 139 cases (25.5%), and bone resection with exarticulation of the temporo-mandibular joint in 11 cases (2.0%). For the most common tumor entity, oral squamous cell carcinoma, the following T-stages (pT) according to the TNM classification were documented postoperatively: pTis (*n* = 15; 2.7%), pT1 (*n* = 211; 38.6%), pT2 (*n* = 132; 24.1%), pT3 (*n* = 75; 13.7%), pT4 (*n* = 75; 13.7%). In 8 cases (1.5%), no tumor (pT0) could be detected postoperatively. The analysis of the neck dissection slides showed the following N-stages (pN): pN0 (*n* = 333; 60.9%), pN1 (*n* = 68; 12.4%), pN2 (*n* = 83; 15.2%), pN3 (*n* = 23; 4.2%). In order to avoid small numbers of cases in the analysis, stages pN2a, pN2b and pN2c and stages pN3a and pN3b were subsumed under stage pN2 and pN3 respectively. In the majority of cases, there was no clinical evidence of distant metastasis (cM0 (*n* = 493; 90.1%)). A cM1 stage was present in 18 cases (3.3%). In terms of reconstruction, 240 local flaps (44.0%), 61 regional (11.2%), and 245 microvascular flaps (44.8%) were performed. Overall, operations averaged 409.5 ± 185.6 min. The extent of postoperative monitoring was determined during preoperative anesthesiologic visit. In some cases, routinely performed monitoring within intermediate care units (IMC) for 24 h had to be upgraded to intensive care unit (ICU) monitoring. This was necessary in 94 cases and was mostly determined preoperatively (*n* = 50, 53.2%). In 7 cases (7.5%) the indication was made intraoperatively, in 37 cases (39.4%) postoperatively, mostly due to medical complications that had occurred.

### The tumor risk score (TRS)

**Fig. 1:**
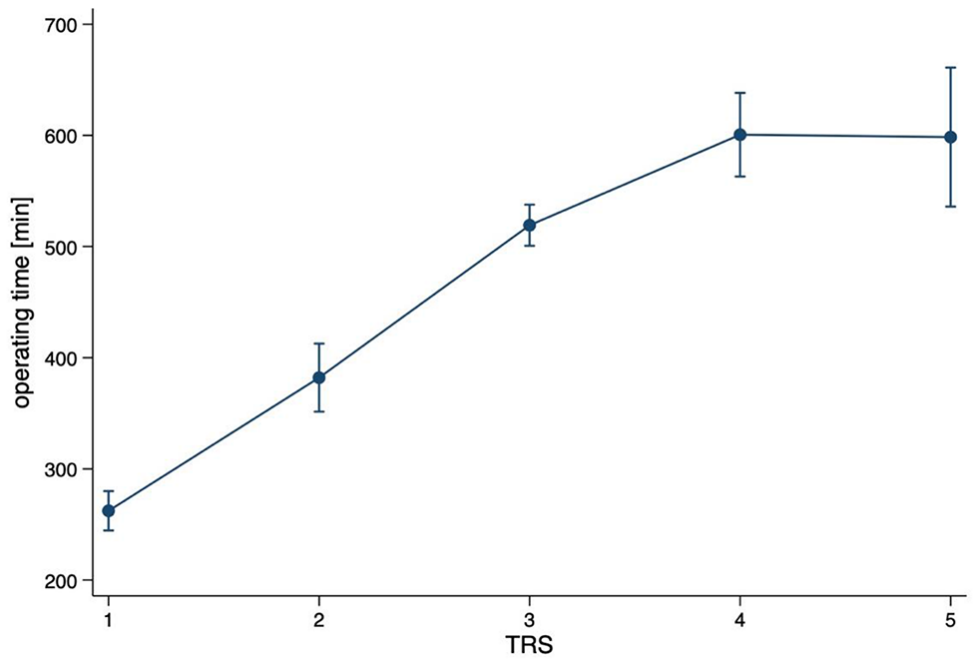
Predictive margins with 95% confidence intervals for operating time [min] associated with TRS (Spearman’s rho 0.72; *p* < 0.001)

**Fig. 2:**
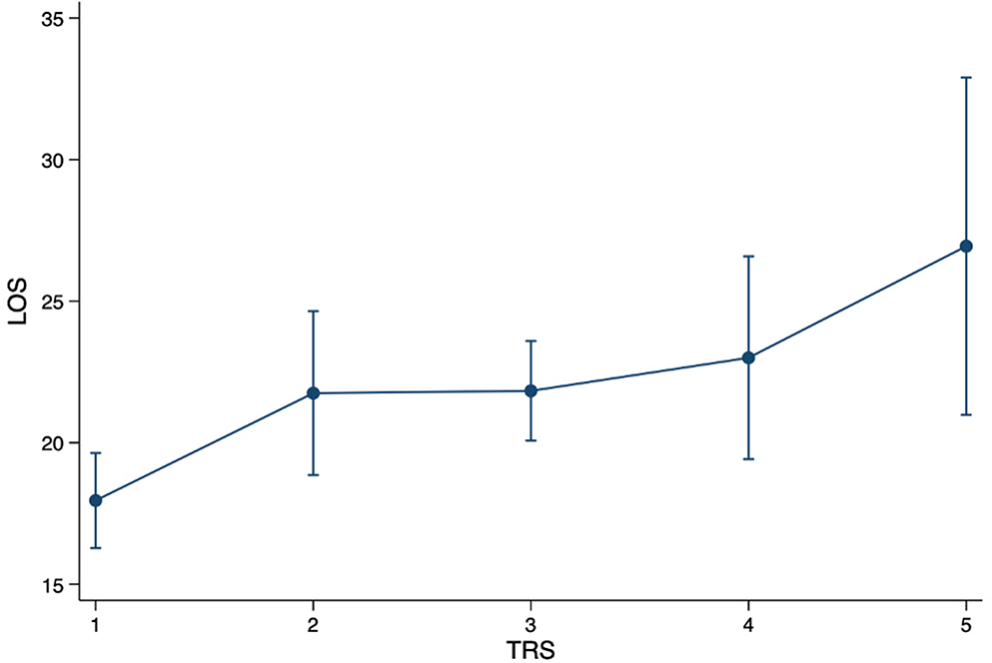
Predictive margins with 95% confidence intervals for total hospital length of stay (LOS) [days] associated with TRS (Spearman’s rho 0.28; *p* = 0.001)

Our newly created Tumor Risk Score (TRS) ranges between 1 and 5 for each surgical tumor intervention, depending on tumor size, location, and level of surgical invasiveness (TRS 1 (*n* = 214 (39.1%)); TRS 2 (*n* = 72 (13.2%)); TRS 3 (*n* = 195 (35.7%)); TRS 4 (*n* = 47 (8.5%)); TRS 5 (*n* = 17 (3.1%))) (Table 1). TRS scores averaged 2.23 (± 1.15). The TRS statistically significantly correlates with surgical duration (Spearman’s rho 0.72; *p* < 0.001) **(**Fig. 1**)** and total LOS (Spearman’s rho 0.28; *p* = 0.001) (Fig. 2). With every gain in TRS, total LOS increased by 9.3% (95%CI 4.7–13.9%; *p* < 0.001) or 1.9 days (95%CI 1.0-2.8 days; *p* < 0.001).

Since tumor size and localization were integrated in the design of the TRS, the correlation with the postoperative T- and N-stages was considered for validation. Correspondingly, there was a statistically significant correlation between the TRS and the pT- (Spearman’s rho 0.25; *p* < 0.001) and pN-stages (Spearman’s rho 0.17; *p* < 0.001). Additionally, the pT- and pN-stages were significantly related to LOS. Both pT (Spearman’s rho 0.40; *p* < 0.001) and pN (Spearman’s rho 0.34; *p* < 0.001) statistically significantly correlate with total LOS. With every increase in pT-stage, total LOS increased by 18.5% (95%CI 13.4–23.6%; *p* < 0.001) or 3.7 days (95%CI 2.8–4.7 days; *p* < 0.001). The trend is similar with the N-stage. With every increase in pN, total LOS increased by 15.7% (95%CI 9.8–21.7%; *p* < 0.001) or 3.2 days (95%CI 2.1–4.4 days; *p* < 0.001).

### Prediction of prolonged IMC/ICU-stay

**Fig. 3:**
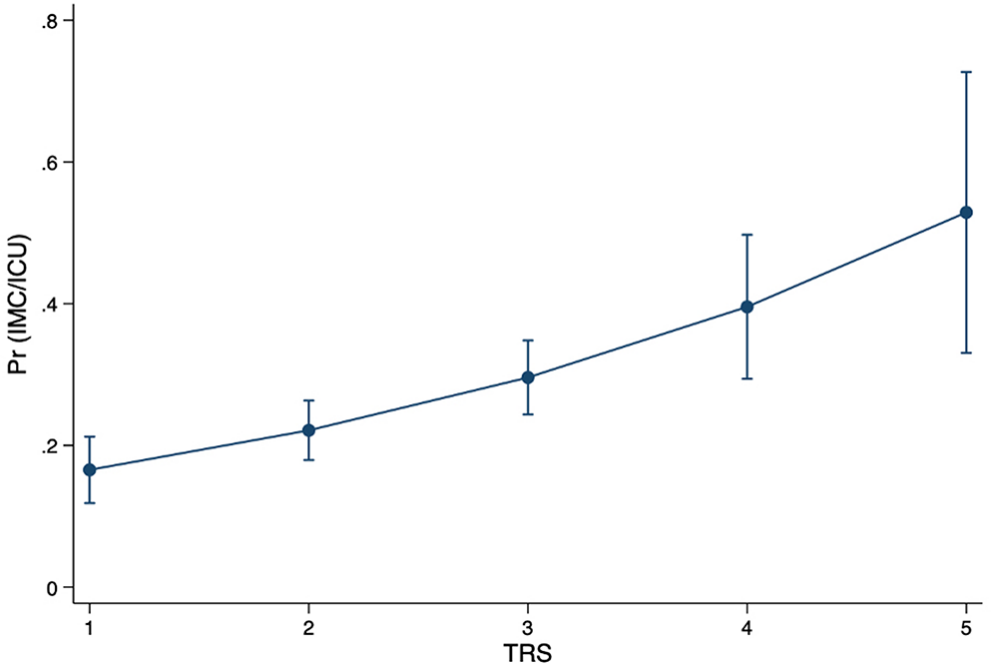
Predictive margins with 95% confidence intervals for the probability of prolonged (> 24 h) intermediate care (IMC) or intensive care unit (ICU) utilization associated with TRS (*p* < 0.001)

Length of stay in the ICU ward was also predicted statistically significant by the TRS. For each increase in TRS by one score point, the LOS in the ICU increased by 0.33 days (95%CI 0.12–0.54 days; *p* = 0.002). The mean postoperative LOS in the IMC ward was 1.29 ± 2.14 days and in the ICU ward 0.66 ± 2.84 days. The probability of a prolonged IMC/ICU stay (Pr(IMC/ICU)) increased significantly (*p* < 0.001) with each increase in TRS (Fig. 3). On average, the probability of a prolonged IMC/ICU stay increased by 32.3% per TRS class (ey/dx; *p* < 0.001).

### Consequences for total LOS and “long-stay patients”

**Fig. 4:**
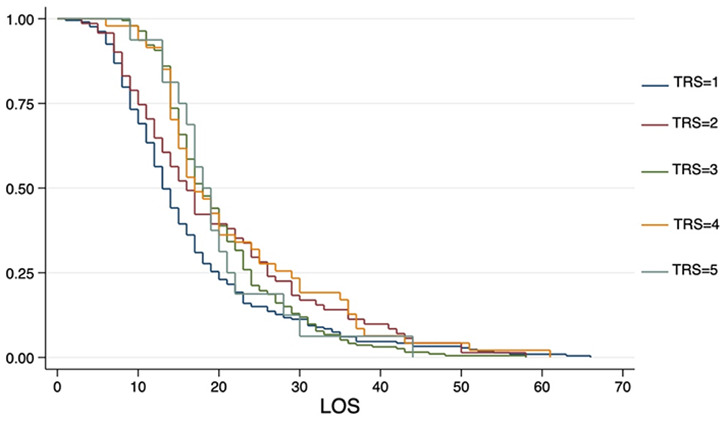
Kaplan-Meier-curve presenting the endpoint of hospital discharge associated with TRS^5^ (logrank *p* = 0.004)

The Kaplan-Meier plot shows the statistically significantly different discharge profiles of patients in the individual TRS groups (logrank *p* = 0.004) (Fig. 4).

**Fig. 5:**
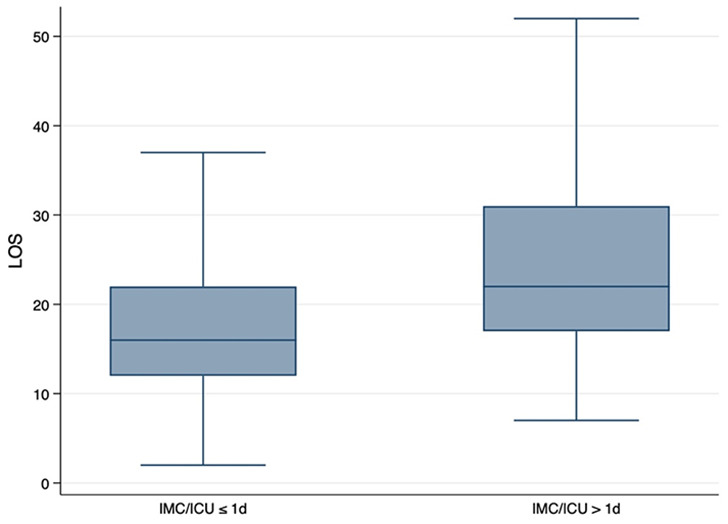
Increase of total hospital length of stay (LOS) [days] associated with prolonged (> 24 h) intermediate care (IMC) or intensive care unit (ICU) utilization (*p* < 0.001)

The consequences of an unplanned extended stay in the IMC or ICU ward also affected the total length of stay in the hospital. When exceeding the scheduled IMC/ICU LOS of 1 day, the total LOS in the hospital increased by 7.7 days (95%CI 5.35–10.08 days; *p* < 0.001) (Fig. 5). This often led to the patients also becoming “long-stay patients” whose upper LOS - depending on the individual case-related DRG - was exceeded and no longer cost-covering. In total, 303 patients (55.4%) stayed longer than the average LOS which was determined by the DRG system for their individual case. 63 patients (11.5%) exceeded the upper limit LOS and became “long-stay patients”. These patients had a mean LOS of 38.8 ± 12.0 days. Of the patient cohort with microvascular reconstruction (*n* = 245), 22 (8.9%) patients were “long-stay patients”, i.e. exceeded the upper limit LOS applicable to their individual case DRG (in the case of DRG “D02A” of 36 days). The median LOS of “long-stay patients” after microvascular reconstruction was 42.2 days.

If the scheduled IMC/ICU LOS of 1 day was exceeded, the patient’s probability for exceeding the upper limit LOS and becoming a “long-stay patient” increased by 70.1% (IRR 1.71; 95%CI 1.02–2.85%; *p* = 0.041).

### The TRS in the context of benchmark risk scores

**Fig. 6:**
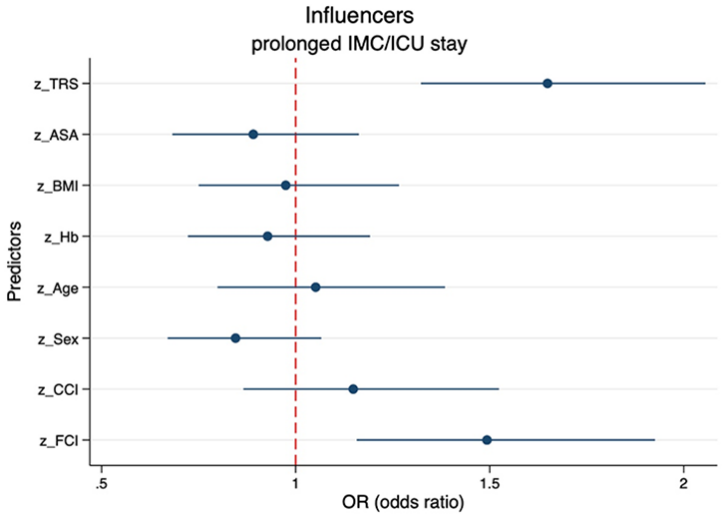
Odds ratios of standardized (z-transformed) preoperative predictor variables on prolonged (> 24 h) intermediate care (IMC) or intensive care unit (ICU) utilization. BMI, body mass index; Hb, hemoglobin; CCI, Charlson Comorbidity Index; FCI, functional comorbidity index

Considering other preoperative patient parameters in terms of their predictive power for prolonged postoperative IMC/ICU stay, the TRS performs better than established risk indices such as ASA class or the Charlson Comorbidity Index (CCI). Using logistic regression with standardized (z-transformed) and thus directly comparable variables, the odds ratio for prolonged IMC/ICU stay increases by 65.0% per TRS score (OR 1.65, 95%CI 1.32–2.06; *p* < 0.001) (Fig. 6). A comparable predictor is the Functional Comorbidity Index (FCI) (OR 1.49, 95%CI 1.16–1.93; *p* = 0.002) [10]. Further standardized predictors such as ASA class (OR 0.89, 95%CI 0.68–1.16; *p* = 0.395), age (OR 1.05, 95%CI 0.80–1.38; *p* = 0.72), preoperative BMI (OR 0.97, 95%CI 0.75–1.27; *p* = 0.847) or CCI (OR 1.15, 95%CI 1.16–1.93; *p* = 0.338) played a minor role.

Thus, the TRS is comparable and performed even better than other benchmarked risk scores. The Patient Clinical Complexity Level (PCCL) is an established value for patient complexity and is used for severity estimation in German hospital revenue calculation. It can adjust for resource consumption within a basic DRG group and compensate for additional costs incurred. It is influenced not only by the level of comorbidity, but also by complications and their severity during or after surgery, such as prolonged IMC/ICU stay. Thus, the final PCCL can only be obtained at the time of discharge. In contrast, our TRS is preoperatively available and statistically significantly correlates with the established PCCL score (Spearman’s rho 0.24; *p* < 0.001).

**Fig. 7:**
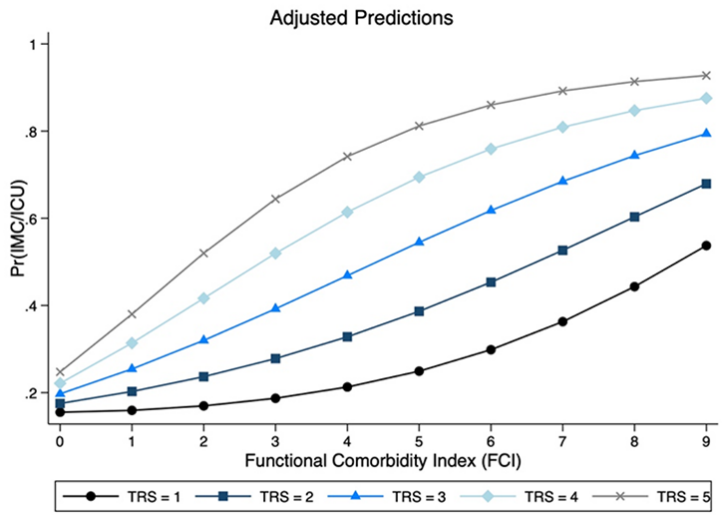
Bidimensional presentation of a three-dimensional risk model with TRS and FCI predicting probability for prolonged (> 24 h) intermediate care (IMC) or intensive care unit (ICU) utilization. FCI, functional comorbidity index

The FCI, a functional comorbidity index that evaluates not only mortality but physical function and functional prognosis, was shown to be a significant and relevant predictor as well (OR 1.49, 95%CI 1.16–1.93; *p* = 0.002). Condensing the logit coefficients of the two predictors TRS and FCI to the exponential function, the probability of prolonged IMC/ICU LOS can be a priori derived in every individual case (Fig. 7).

**Fig. 8:**
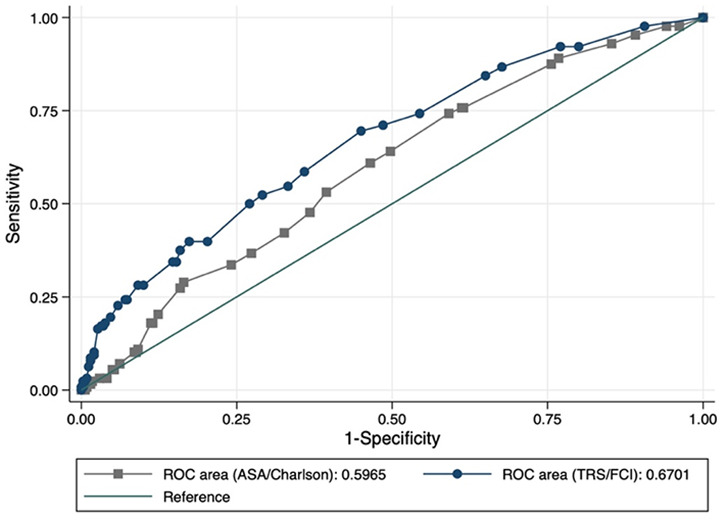
Comparing the goodness of fit of model **a** (ASA, CCI) and model **b** (TRS, FCI) predicting prolonged (> 24 h) intermediate care (IMC) or intensive care unit (ICU) utilization (cutoff-probability 0.2; Bonferroni *p* = 0.031). ROC, receiver operating characteristic

Predictive model power is even better than a model with ASA class and CCI, which are routinely used to assess the peri- and postoperative risk of complications (cutoff-probability ≥ 0.2; Bonferroni *p* = 0.031) (Fig. 8).

## Discussion

Surgical resection and reconstruction remain the leading treatment modalities for head and neck tumors. However, patients often present with significant comorbidities which can lead to complications during or after lengthy surgical procedures and may require a high-care environment for postoperative recovery. In demand for evidence-based clinical care during the era of limited or constrained financial and human resources in hospitals, it is crucial to implement appropriate and standardized triage for immediate postoperative management of these patients.

The classification developed by Saklad et al. in 1941, modified in 1961, and published by the American Society of Anesthesiologists (ASA), was constructed to preoperatively assess overall health of surgical patients and estimate peri- and postoperative complications [11]. In literature, ASA rating correlates with a higher number of postoperative complications after microvascular reconstruction, such as flap failure or overall survival time [7, 12–15]. However, Mücke et al. criticized the ASA-score to be sweeping and nonspecific for the variability of head and neck tumor patients [15]. In our study, ASA score as a standardized predictor (OR 0.89, 95%CI 0.68–1.16; *p* = 0.395) performed worse than the new TRS (OR 1.65, 95%CI 1.32–2.06; *p* < 0.001) in predicting prolonged IMC/ICU stay (Fig. 6). Preoperative health status is often condensed in various comorbidity scores during preoperative anesthesiologic evaluation. In this regard, the Charlson Comorbidity Index (CCI) uses a weighted cumulative index with categorized comorbidities that showed a significant association with postoperative mortality [16]. In major head and neck surgery overall mortality is rare (0.5–1.5%) and commonly due to myocardial infarction, embolism, or pneumonia [17]. Therefore, focusing on mortality as a possible endpoint parameter, rather than on specific complications such as prolonged IMC/ICU stay, may be one of the reasons why CCI appears to be less relevant in our study population as well (OR 1.15, 95%CI 1.16–1.93; *p* = 0.338) (Fig. 6). The authors that created the Functional Comorbidity Index (FCI) hypothesized that comorbidities associated with physical function are distinct from those primarily associated with postoperative mortality [10]. Therefore, FCI performs better in predicting physical function than indices predicting postoperative mortality as an outcome [18, 19]. For predicting prolonged IMC/ICU stay, this is both clinically relevant and statistically significant (OR 1.49, 95%CI 1.16–1.93; *p* = 0.002) (Fig. 6).

In order to account for procedure-specific risk, the ESA/ESC guidelines classify surgical risk by the incidence of cardiovascular-related lethality or the incidence of non-fatal myocardial infarction within 30 days regardless of any patient comorbidities. Incidences < 1% are assessed as low, between 1% and 5% as intermediate, and > 5% as high intervention-specific risk [20]. The Surgical Risk Classification according to John Hopkins, which is also widely used, graduates five groups according to the increasing degree of invasiveness of the surgical procedure. It is interdisciplinary and does not discriminate among the surgical spectrum of different surgical disciplines [21]. However, not only do oral and maxillofacial surgical procedures differ from one another in terms of invasiveness, complexity, blood loss, duration of surgery etc., even tumor surgery in the head and neck region itself offers a wide range of procedures. These can only be stratified vaguely by means of a broad and interdisciplinary score. Especially colleagues from other disciplines sometimes lack differentiation criteria for enhanced resource planning. Ex post, a more precise differentiation can be achieved using the operating time. However, this information is not available preoperatively and cannot be used for predictive risk evaluation and resource allocation. In general, our partners in anesthesia often regard a routine 24-hour monitoring in the intermediate or intensive care unit as necessary and safe [22, 23].

In order to preoperatively calculate the individual probability of a prolonged IMC/ICU stay we combined the two relevant predictors TRS and FCI in order to enable an intervention-specific risk prediction (TRS) in addition to the patient-specific risk constellation for complications (FCI) (Fig. 7). If the TRS and FCI coefficients of the logit function are condensed to the exponential function, the probability PR (IMC/ICU prolonged) can be directly derived [24]:

$$\eqalign{{\rm{PR}}\,\left( {{\rm{IMC/ICU\, prolonged}}} \right){\rm{ = }} & {\rm{ exp}}\,\left( {{\rm{0}}{\rm{.44}}\,{\rm{*}}\,{\rm{TRS}}\,{\rm{ + }}\,{\rm{0}}{\rm{.29}}\,{\rm{*}}\,{\rm{FCI}}\,{\rm{-}}\,{\rm{2}}{\rm{.7}}} \right)\, \cr & {\rm{/}}\,\left( {{\rm{1 + exp}}\,\left( {{\rm{0}}{\rm{.44}}\,{\rm{*}}\,{\rm{TRS}}\,{\rm{ + }}\,{\rm{0}}{\rm{.29}}\,{\rm{*}}\,{\rm{FCI}}\,{\rm{-}}\,{\rm{2}}{\rm{.7}}} \right)} \right){\rm} \cr}$$

If, for example, a patient with cT2cN0 squamous cell carcinoma of the mandible and anterior floor of the mouth (2 regions = TRS 1) undergoes tumor resection with continuity-preserving mandibular bone resection and reconstruction with radial forearm flap (surgical procedure = TRS + 3), a total TRS of 4 results. With regard to comorbidities, the sample patient presents with osteoporosis and peripheral vascular disease, resulting in an FCI of 2 points. The sample patient thus has a probability of remaining in the IMC/ICU ward for more than 1 day of:

Pr (IMC/ICU prolonged) = exp(0.44 * 4 + 0.29 * 2–2.7) / (1 + exp(0.44 * 4 + 0.29 * 2–2.7)) = 0.411, or 41.1%. With a cutoff value of 0.2, this model of TRS and FCI (ROC area (TRS/FCI) = 0.6701) achieves a better prediction than a prediction model of ASA class and CCI (ROC area (ASA/Charlson) = 0.5965) (*p* = 0.031) (Fig. 8).

If patients at a high risk of a prolonged IMC-/ICU stay could be identified preoperatively, additional safety measures could be taken or the surgeon may, in some cases, even select an alternative reconstructive strategy. This predictability index could be used in the preoperative setting to improve patient counseling, as well as postoperative organization and treatment algorithms. While most components of risk prediction are not reversible, they do raise awareness among the medical team and can provide patients with realistic expectations regarding their surgery and postoperative course.

Additionally, the risk of postoperative complications does not necessarily equate the need for critical care services. A report from Hong Kong [23] concluded that many anesthesiologists recommend generalized monitoring for 24 h in the ICU after major head and neck tumor surgery often based on defensive medical practice or factors other than medical necessity [25]. In several parts of the world, different surgical specialties perform head and neck tumor surgery either alone or as part of a multidisciplinary surgical team that may include oral and maxillofacial surgeons (OMF), general surgeons, plastic and reconstructive surgeons (PRS), and ear-nose-throat surgeons (ENT). Each of them may contribute a different perspective and expertise to the care and treatment [22]. The British Association of Head and Neck Oncologists asked 253 surgeons from ENT, OMF and PRS about their use of intermediate and intensive care facilities after head and neck tumor surgery [26]. Regarding microvascular flaps, PRS preferred monitoring in an IMC ward, whereas OMF surgeons tended to use the ICU ward. After tracheostomy most ENT and OMF surgeons felt comfortable with care in a regular ward, whereas PRS preferred the IMC. Over all surgical disciplines, one third of the surgeons would start a surgical tumor resection with microvascular reconstruction even if the monitoring ward of their choice (IMC or ICU) would not be available. The survey suggests that there are different approaches and preferences regarding postoperative care after head and neck tumor surgery not only between different surgical departments nationally and internationally but also within the field of maxillofacial surgery itself. Resources, type of hospital care, staff qualifications, availability of IMC/ICU ward facilities, expertise and workload of (nursing) staff and funding issues may be additional factors influencing the postoperative regimen [27]. Thus, it is a potential limitation of our study that we used individual and hospital-specific criteria defining the outcome of “prolonged IMC-/ICU-stay" > 24 hours. The TRS and our risk prediction model may not be directly applicable to other hospitals.

Lack of standardized monitoring protocols or inaccurate assessment of needs can lead to both under- and overutilization of IMC and ICU wards. Monitoring of microvascular flaps can be a trigger for IMC or ICU transfer in some hospitals due to trained nursing staff and more favorable patient-nurse-ratios [28]. According to a survey of plastic surgeons up to 61% of microsurgical centers in the United States prefer to monitor their free flap reconstructions in the ICU. ICU utilization is a major component of hospital resource costs in the United States with significant financial implications. Cornejo et al. [28] found an average length of ICU stay after free flap reconstruction in head and neck surgery of 5.8 ± 0.5 days. Patients with postoperative complications had longer ICU stays compared to patients without complications (8.8 ± 13.4 versus 5.6 ± 15.3 days, *p* = 0.24). In our study, the difference in the discharge profiles of patients in the individual TRS groups was statistically significant (logrank *p* = 0.004) (Fig. 4). When the regular IMC/ICU length of stay of 1 day was exceeded, the total hospital length of stay increased by 7.7 days (95%CI 5.35–10.08 days; *p* < 0.001). In Germany, this fact is already taken into account in revenue calculation. Comorbidities as well as planned and unexpected procedures trigger a complex calculation of a case-specific patient comorbidity complexity level (PCCL), which impacts the case-specific revenue. Likewise, the lower, average and upper hospital length of stay is calculated individually and influenced by PCCL score. Thus, PCCL regulates resource consumption within individual cases and can compensate for additional costs. Comparing the well-established PCCL as a benchmark predictor to our TRS shows a statistically significant correlation (Spearman’s rho 0.24; *p* < 0.001). Unlike PCCL, our TRS is already available at the time of hospital admission which enables a priori risk stratification and resource scheduling.

In Germany, OMF tumor resection and microvascular reconstruction currently generates a total revenue of EUR 23,583, a lower limit LOS of 6 days, an average LOS of 20.1 days and an upper LOS of 36 days. As we did not want to compare absolute numbers in total LOS, we calculated the proportional LOS considering the actual LOS and case-specific mean LOS of patients with the same spectrum of comorbidities and surgical procedures. We thus created the parameter “long-stay patients” if the proportional LOS was larger than the case-specific upper limit LOS. If a patient exceeds the upper limit LOS the daily revenue is no longer cost-covering. If the regular IMC/ICU LOS of 1 day was exceeded, the probability for also exceeding the upper limit LOS and becoming a “long-stay patient” increased by 70.1% (IRR 1.71; 95%CI 1.02–2.85; *p* = 0.041). In the German Health Care System exceeding the upper limit LOS is no longer cost-effective, besides the fact that utilization of the ICU is a cost-increasing factor itself. Especially in case of additional postoperative complications the increase in total revenue can remain insufficient while simultaneously more resources are consumed. This jeopardizes cost-effective patient care especially at hospitals providing specialized and maximum care. Already in 2007, Jones et al. [29] urged to directly focus on reducing peri- and postoperative medical complications when striving for cost-effectiveness of microsurgical reconstruction in head and neck tumor surgery. In this context it has to be evaluated whether enhanced care by skilled stuff on general surgical wards can reduce ICU utilization and enable a more targeted allocation of the resource “ICU monitoring” without compromising patient care [22, 30]. According to Haddock et al. [31] the cost per ICU bed in the US amounts to approximately $2,360 per day compared to $900 in a non-ICU setting. In our institution, the cost of ICU monitoring is EUR1,200/day, while a total amount of EUR2,300 for financing the patient’s ICU stay is included in the total case revenue of tumor resection with microvascular reconstruction. Therefore, ICU monitoring is not modeled to cover costs after the 3rd day. In this regard, our index helps both surgeons and anesthesiologist to stratify the risk of a prolonged IMC/ICU stay during the preoperative visit and to adjust resource allocation in specific situations.

Finally, our TRS helps to attribute complication rates and enable a fair comparison of the quality of care in different hospital settings through risk-related adjustments. In 2016, a new law to reform the structures of hospital care came into force in Germany. This mandates the concept of quality-based reimbursement elements in certain specialties. To improve outcome quality additional surcharges and penalty fees for good and inadequate quality of care are being developed. Therefore, specialized and established quality indicators are used. In order to enable a comparative evaluation of the results of different hospitals, the risks of the different patient groups must also be taken into account. It is crucial to compare quality measures regardless of occurring complications on external factors (patient complexity, complexity of surgery), so that an objective risk adjustment can be assumed. This is particularly relevant for hospitals providing maximum care as they cannot perform risk selection [32, 33].

A potential limitation of our study is its retrospective design that can account for observer bias. We are constantly collecting prospective data on patients with head and neck tumor surgery to further validate our index. Additionally, we would like to add that established medical scores such as the ASA classification or the Charlson score have their rightful place in preoperative risk analysis, especially in an inhomogeneous patient collective. Thus, our index certainly cannot be regarded as an exclusive tool, but can be used as a supplementary instrument in the prediction of resource-intensive patient courses, particularly in maximum care hospital settings. Furthermore, the index was created based on the specific circumstances of our hospital and our patient population, whether it can be transferred to other clinics or to countries with other health care systems has yet to be studied.

## Data Availability

No datasets were generated or analyzed during the current study.
